# Nursing at the Royal Infirmary of Edinburgh

**Published:** 1921-01-29

**Authors:** 


### Nursing at the Royal Infirmary of Edinburgh.

THE leading fact about nursing prospects in Edinburgh is the revival of applications from candidates. At the Royal Infirmary during the past year the total numbered 523, out of which 101 were selected to fill vacancies on the staff. Most training-schools down south would be considerably relieved if they could get five candidates to choose from for each vacant place. The recent report of the Royal Infirmary is somewhat reduced in size, but, as usual, careful statistics are given of the training-school for nurses. The daily average of nurses on the staff numbered 312, the highest number on any given day being 325. Three sisters retired on pensions, Sister E. M. Herriot, after twenty-seven years; Sister H. Christie, after twenty-three years; and Sister E. Caldwell, after seventeen and a-half years' service. The resignation of Miss M. C. Herriot, who relinquished her post as Senior Assistant to the Lady Superintendent of Nurses, after a period of twenty-nine years' service, marks something of an epoch in the institution, which is famed for its power of retaining sisters devoted to its interests. Among those who completed their training or passed out of the infirmary during the year, the largest number—twenty-two—left to take up private nursing. Thirteen took posts in other hospitals, eleven went home, three left to be married, nine to take maternity training, one to Q.V.J.I., and one to go abroad. None are reported as taking up public health work, unless those who were taking midwifery training were preparing for this branch of their profession. Of the probationers in training two left to be married, two on account of home circumstances, and two for reasons of health.

The managers have authorised an increase of thirty nurses to the staff in order to secure a reduction in the hours on duty. With a view to providing more accommodation for the nursing staff Ward 22 has been fitted with cubicles. By means of this extension the hours on duty can be brought down to fifty-six in the week, a reform which has been long desired. There will be no difficulty here in securing the extra probationers required, and we trust the new system will shortly be in perfect working order. It is very satisfactory to learn that the health of the nurses has been exceptionally good throughout the year, the daily percentage off duty through illness, including infectious diseases and convalescence, being only 2.6. This should dispose of any notion that the work of a nurse, even when engaged for considerably more than fifty-six hours a week, is of a nature to break down her health. Taking it all in all, the daily life in a well-managed institution such as the Royal Infirmary, in close association with some 800 persons suffering from divers diseases, is one of the healthiest which can be chosen. It would be interesting to take at haphazard 300 ordinary young women of leisure, ranging in age from twenty-two to twenty-six, and reckon up the daily percentage of illness which occurred among them. It would almost certainly compare very unfavourably with the illness average of the nursing staff at the Royal Infirmary. But the longer hours off duty, even if not needed for improvement of health, are indispensable for the widening of the mind and the quickening of the intelligence during the training years.

